# Determination of Lekethromycin, a Novel Macrolide Lactone, in Rat Plasma by UPLC-MS/MS and Its Application to a Pharmacokinetic Study

**DOI:** 10.3390/molecules25204676

**Published:** 2020-10-13

**Authors:** Hongzhi Xiao, Pan Sun, Jicheng Qiu, Jianzhong Wang, Lei Yan, Suxia Zhang, Xingyuan Cao

**Affiliations:** 1 Department of Veterinary Pharmacology and Toxicology, College of Veterinary Medicine, China Agricultural University, Beijing 100193, China; xiaohongzhi1992@163.com (H.X.); sunpan@cau.edu.cn (P.S.); qujicheng2015@163.com (J.Q.); jianzhongwang@cau.edu.cn (J.W.); suxia@cau.edu.cn (S.Z.); 2 Laboratory of Quality & Safety Risk Assessment for Animal Products on Chemical Hazards (Beijing), Ministry of Agriculture, Beijing 100193, China; 3 Henan Pulike Biological Engineering Co., Ltd., Luo Yang, Henan 471000, China; yanlei@pulike.com; 4 Key Laboratory of Detection for Veterinary Drug Residue and Illegal Additives, Ministry of Agricultural, Beijing 100193, China

**Keywords:** UPLC-ESI-Orbitrap-MS, lekethromycin, rats, pharmacokinetic

## Abstract

Lekethromycin, a new macrolide lactone, exhibits significant antibacterial activity. In this study, a reliable analytical ultrahigh-performance liquid chromatography electrospray ionization quadrupole Orbitrap high-resolution mass spectrometry (UPLC-ESI-Orbitrap-MS) method was established and validated for the detection of lekethromycin in rat plasma. After a simple acetonitrile (ACN)-mediated plasma protein precipitation, chromatographic separation was performed on a Phenomenex Luna Omega PS C18 column (30 × 2.1 mm i.d. particle size = 3 μm) conducted in a gradient elution procedure using 0.5% formic acid (FA) in ACN and 0.5% FA in water as the mobile phase pumped at a flow rate of 0.3 mL/min. Detection was carried out under positive electrospray ionization (ESI+) conditions in parallel reaction monitoring (PRM) mode with observation of *m/z* 804.5580 > 577.4056 for lekethromycin and 777.5471 > 619.4522 for gamithromycin (internal standard, IS). The linear range was 5–1000 ng/mL (r^2^ > 0.99), and the lower limit of quantification (LLOQ) was 5 ng/mL. The intra- and inter-day precision (expressed as relative standard deviation, RSD) values were ≤7.3% and ≤6.3%, respectively, and the accuracy was ≥90% ± 5.3%. The mean extraction recovery RSD valWeue was <5.1%. Matrix effects and dilution integrity RSD values were <5.6% and <3.2%, respectively. Lekethromycin was deemed stable under certain storage conditions. This fully validated method was effectively applied to study the pharmacokinetics of lekethromycin after a single intravenous administration of 5 mg/kg in rats. The main pharmacokinetic parameters were T_1/2λz_, CL_obs and V_Z__obs were 32.33 ± 14.63 h, 0.58 ± 0.17 L/h/kg and 25.56 ± 7.93 L/kg, respectively.

## 1. Introduction

Macrolides are an important class of antibiotics widely prescribed for the treatment of human and veterinary infectious disease [1,2]. Many macrolide antibiotics are produced by various *Streptomyces* strains and some other bacteria, such as *Arthrobacter* spp [3,4]. The majority of macrolide antibiotics are composed of a distinctive macrocyclic lactone ring to which one or more cladinose-neutral and desosamine-amino sugar residues are linked via glycosidic bonds [3,5]. The most commonly used macrolides can be conventionally categorized based on the size of the macrocyclic lactone ring into groups of either 14-, 15- or 16-membered ring macrolides [5,6,7]. In general, all macrolide antibiotics act mainly on Gram-positive cocci and display only limited potency against some Gram-negative bacteria [5,7]. Additionally, they are also particularly effective in the treatment of various *Mycoplasmas*, *Rikettsia*, *Legionella pneumophila*, *Haemophilus influenzae* and *Chlamydia* spp. [4,5,8,9]. Most notably, macrolide antibiotics have become an important alternative for patients allergic to penicillin [3,4]. The antibacterial mode of action of most macrolides is inhibition of protein synthesis by reversible binding to the bacterial 50S ribosomal subunit [6,10,11,12,13,14,15]. Recently, it was reported that macrolides also have good anti-inflammatory activity, resulting from down-regulation of the production and secretion of proinflammatory cytokines, such as TNF-α, IL-1, IL-6, IL-8 and ENA-78 [16,17,18,19]. All of these properties have led to the remarkable success of macrolides in the treatment of bacterial infectious diseases, as well as chronic inflammatory disease [3,20,21].

However, following broad use of macrolide antibiotics over the past several decades, the emergency of resistance was reported by many countries in some of the common pathogens, such as *Treponema pallidum* [22,23,24], *Mycoplasma pneumoniae* [25] and group A *Streptococci* [26]. Consequently, in order to address increasing macrolide resistance, research into the development of new antimicrobial macrolides is of great necessity [15].

**Figure 1:**
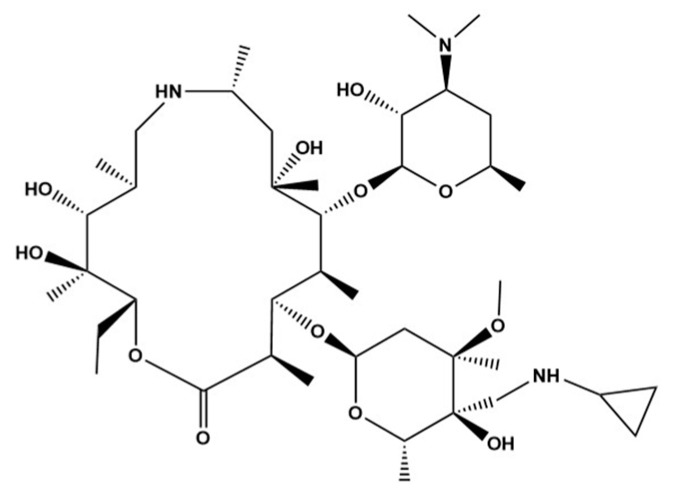
Chemical structure of lekethromycin.

Recently, lekethromycin (Figure 1), a new macrolide, was semi-synthesized and characterized by infrared spectrometry (IR), high resolution mass spectrometry (HR-MS), nuclear magnetic resonance spectroscopy (NMR) and single crystal X-ray diffraction analysis. The molecular structure of lekethromycin is similar to those of azithromycin and tulathromycin. We have obtained the granted patent in China (CN 103965273 B) for the chemical synthesis and antibacterial bioactivity of the compound, which is currently in preclinical trials [27]. Since pharmacokinetic information has an important role in many vital aspects of new drug discovery and development [28,29,30], development of a fast and accurate method for the determination and monitoring of lekethromycin in rat plasma is urgently required. To the best of our knowledge, there are no prior publications regarding method establishment and validation for the measurement of lekethromycin in biological samples. Therefore, the goal of this present study was to explore and develop a sensitive, accurate and rapid quantification method for the determination of lekethromycin in rat plasma. The established method satisfied full validation criteria and was successfully applied to the pharmacokinetic study of lekethromycin in rat plasma after intravenous administration of a single dose of 5 mg/kg.

## 2. Material and Methods

### 2.1. Chemicals and Reagents

The lekethromycin standard (batch no. D20170101, purity ≥97%) was obtained from Henan Pulike Biological Engineering Co., Ltd (Luoyang, Henan, China). An authentic reference standard of gamithromycin (CAS 145435-72-9, purity ≥97%, internal standard, IS) was purchased from Sigma-Aldrich (St. Louis, MO, USA). LC-MS grade methanol (MeOH), acetonitrile (ACN) and formic acid (FA) (98% purity) were supplied by Fisher Scientific (Pittsburgh, PA, USA). Propylene glycol, PEG400 and dimethyl sulfoxide (DMSO) were of analytical grade and were purchased from Sinopharm Chemical Reagent Beijing Co., Ltd (Beijing, China). Ultrapure water was obtained from a Millipore Milli-Q purification system (Bedford, MA, USA). Blank rat plasma with EDTA-k2 anticoagulant was centrifuged at 4000× *g* for 10 min and was stored at −80 °C until analysis.

### 2.2. Preparation of Stock Solutions, Quality Control Samples and Standard Curves

The standard stock solutions of both lekethromycin and IS at 1.00 mg/mL were obtained by dissolving the respective powders with MeOH in a volumetric flask. The corresponding working standard solutions were prepared by appropriate dilutions of the stock solution with MeOH to provide concentrations of 50, 200, 500, 2000, 5000 and 10,000 ng/mL of lekethromycin and 100 ng/mL of IS. The quality control (QC) working solutions were prepared by diluting a separate lekethromycin standard stock solution with MeOH to give final concentrations of 150, 2000 and 8000 ng/mL. All prepared stock and working solutions were stored at −20 °C until use.

To prepare calibration standard curves, aliquots (20 μL) of the working standard solutions were spiked into 200 μL of blank pooled plasma to obtain final concentrations of 5, 20, 200, 500 and 1000 ng/mL for lekethromycin and 10 ng/mL for the IS. QC samples were prepared using the same procedure at the ultimate low, medium and high concentrations (15, 200 and 800 ng/mL).

### 2.3. LC-MS/MS Analysis

The analysis was performed using an ultimate 3000 ultrahigh-performance liquid chromatography (UPLC) chromatography system coupled to a Q-Exactive orbitrap high-resolution mass spectrometer, both from Thermo Scientific (Pittsburgh, PA, USA). Xcalibur software 2.3.1 (Thermo Fisher, Pittsburgh, PA, Scientific, USA) was used to control the instruments and for data acquisition and processing. Chromatographic separation was achieved on a Phenomenex Luna Omega PS C18 column (30 × 2.1 mm i.d. particle size = 3 μm), with the flow rate at 0.3 mL/min at 35 °C. Samples were maintained at 7 °C in the autosampler. The mobile phase consisted of 0.5% aqueous FA (A) and 0.5% FA in ACN (B). The optimized gradient was as follows: 5% B at 0–0.5 min; 5%–90% B at 0.5–1.5 min; 90% B at 1.5–2.0 min; 90%–5% B at 2.0–2.2 min; 5% B at 2.2–3.5 min.

Mass detection was conducted by parallel-reaction monitoring (PRM) in positive ion electrospray ionization interface (ESI) mode. The optimized ESI source parameters were as follows: spray voltage 3.2 kV; automatic gain control (AGC) target 1 × 10^5^; capillary temperature 320 °C; resolution 17,500; CE 40 eV; vaporizer temperature 400 °C; maximum injection time (IT) 200 ms; sheath gas flow rate 4.58 L/min; auxiliary gas flow rate 10.75 L/min; isolation window 2.0 *m/z*. The PRM precursor-to-product transitions for lekethromycin and IS were *m/z* 804.5580 > 577.4056 and *m/z* 777.5471 > 619.4522, respectively.

### 2.4. Animals

Five male Sprague-Dawley rats weighing 210–240 g were purchased from Animals-Science Co., Ltd (Beijing, China). All of the animals were acclimatized in an environmentally controlled breeding room (humidity 50%–60%, temperature 19–26 °C, 12 h light/dark cycle) for 7 days, with free access to food and water. Before the experiment, the rats were fasted for 12 h, but water was freely accessible. The animal experiments were performed in accordance with the Guide for Care and Use of Laboratory Animals and approved by the Review Committee of Animal Care and Use of China Agricultural University (14408-19-R-026) (Beijing, China).

### 2.5. Sample Preparation

Frozen plasma samples were thawed at room temperature in advance for approximately 20 min, and then the plasma was vortexed. An accurately measured aliquot (200 μL) of plasma was transferred to a blank Eppendorf microcentrifuge tube, followed by the addition of 20 μL IS working standard solution (100 ng/mL of gamithromycin in MeOH). After vortexing for 1 min, 600 μL of ACN was added to precipitate the protein. The plasma sample was vortexed for 1 min and then centrifuged at 12,000× *g* at 4 °C for 20 min. The supernatant was pipetted into a clean tube, evaporated to dryness under a nitrogen stream without heating, reconstituted with 400 μL ACN:1% FA (1:9, *v*/*v*) and then centrifuged at 12,000× *g* for 20 min at 4 °C. Finally, a 5 μL aliquot of supernatant was injected into the LC-MS/MS system for analysis.

### 2.6. Method Validation

The method validation was performed according to the United States Food and Drug Administration (FDA) and European Medicines Agency (EMA) guidelines for bioanalytical method validation [31,32].

The specificity of the proposed procedure was estimated by comparing chromatograms of blank rat plasma samples from at least six different sources with blank samples that had been spiked with lekethromycin and IS at concentrations corresponding to the lower limit of quantification (LLOQ). The lekethromycin should be completely separated, and no potential interference peaks detected at the retention times of lekethromycin or IS.

The sensitivity was assessed by the lower limit of quantification (LLOQ). The LLOQ is usually expressed as the lowest concentration on the calibration curve with an *S/N* value of more than 10 and a relative standard deviation (RSD) within 20%.

Calibration curves for lekethromycin were established by correlating the peak area ratios (lekethromycin/IS) to the corresponding lekethromycin nominal concentrations covering the expected range of 5–1000 ng/mL. Linear regression analysis using the 1/x^2^ weighting factor was performed to obtain the correlation coefficient (R). The calculated standard concentration should be within 15% of the theoretical concentration, and R^2^ was >0.99. The carryover effect was evaluated by injecting blank plasma samples after the upper limit of quantification (ULQ) samples.

The accuracy and precision of the method were investigated by analyzing QC samples at three concentration levels. The analyzed concentrations were 15 ng/mL (low quality concentration, LQC), 200 ng/mL (medium quality concentration, MQC) and 800 ng/mL (high quality concentration, HQC). For the calculation of intra- and inter-day accuracy and precision, five replicate QC samples at each concentration were analyzed in one day and on three consecutive days, respectively. Accuracy was calculated as the deviation of the measured concentrations from the theoretical concentration of the QC samples, expressed as a percentage. Precision meant the closeness of individual values of the analyte and was expressed as RSD. The accuracy and precision should be 85%–115% and less than 15%, respectively.

Matrix effect (ME) and recovery were estimated at three concentration levels (15, 200 and 800 ng/mL). For the ME effect, six lots of blank samples were processed as described previously and then spiked with QC samples post extraction. The ME was evaluated by comparing the peak area ratios of the post-extraction spiked QC samples with those of the mobile phase solution at the same concentrations. The IS normalized ME also was calculated by dividing the ME of the lekethromycin by that of the IS. Recovery of lekethromycin and IS was determined by comparing the peak areas of regularly prepared QC samples with those of post extraction blank samples spiked at the equivalent concentration.

To evaluate the dilution effect, 2 mg/mL QC plasma samples were diluted two-, ten- and a hundred-fold with blank rat plasma, repeated six times at each dilution factor, to yield final concentrations of 1000, 200 and 20 ng/mL, respectively. Dilution integrity was considered to be acceptable if the accuracy and precision of the diluted samples were within ±15% and RSD <15%, respectively.

The stability of lekethromycin in rat plasma was assessed by analyzing QC samples (*n* = 3) at LQC, MQC and HQC concentrations after three complete freeze-thaw cycles and after storage at 25 °C for 24 h (short term stability), at −20 °C for two months (long term stability) and at 7 °C for 8 h in the autosampler (autosampler stability). The stability of lekethromycin in the stock solution (1 mg/mL) stored at −20 °C for three months was also assessed.

### 2.7. Application to a Pharmacokinetic Study

Following overnight fasting, rats were dosed intravenously with 5 mg/kg of lekethromycin formulated in 45% PEG400, 10% DMSO and 45% propylene glycol. Blood samples (~0.25 mL) were collected from the retinal venous plexus into heparin-containing tubes at different time intervals: pre-dose, 0.083, 0.25, 0.5, 1, 2, 3, 6, 8, 10, 12, 24, 48, 72, 96, 120, 144, 192 and 240 h after dosing. Plasma samples were immediately separated by centrifugation of the blood at 4000× *g* for 10 min and were stored at −80 °C before analysis. The main pharmacokinetic parameters, including plasma clearance (CL), the maximum concentration (C_max_), area under the plasma concentration-time curve from 0 to the last point of the measured concentration (AUC_last_), elimination half-life (T_1/2λz_) and mean residence time (MRT), were analyzed and calculated using the non-compartmental model in WinNonlin 6.4 pharmacokinetic software (Pharsight Corporation; Mountain View, CA, USA). Pharmacokinetic data are presented as mean ± SD (standard deviation, SD).

## 3. Results and Discussion

### 3.1. UPLC-Mass Spectrometry Optimization

This study established, for the first time, an ultra-performance-liquid chromatography system coupled to a Q-orbitrap mass spectrometry detector for the determination of lekethromycin in rat plasma.

To optimize the mass spectrometer parameters, we compared the positive and negative ionization detection modes and found that the signal response in positive-ion mode was significantly better than that in negative-ion mode. Parallel reaction monitoring is a commonly used detection method and provides strong specificity, satisfactory reproducibility and high sensitivity. Ions at *m/z* 804.5580 and *m/z* 777.5471 were chosen as precursors for Mass Spectrometers Tandem Mass Spectrometry (MS/MS) analysis of lekethromycin and IS, respectively. The most abundant fragment ions of lekethromycin and IS were at *m/z* 577.4056 and 619.4522, respectively. Therefore, the transitions for quantification were *m/z* 804.5580 > 577.4056 for lekethromycin and 777.5471 > 619.4522 for IS. Gamithromycin, a structural analog of lekethromycin, was selected as the internal standard because, like lekethromycin, it is poorly soluble in water. This is essential for chromatographic separation on a C18 column and efficient extraction with ACN. Moreover, the retention time was appropriate and did not interfere with the lekethromycin peak.

**Figure 2:**
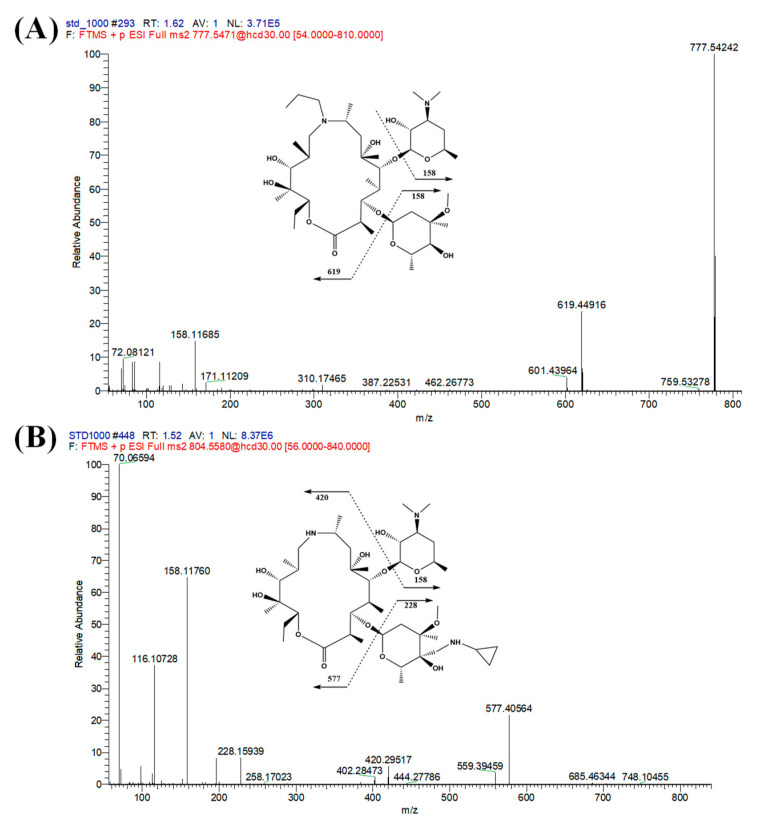
The mass spectra of gamithromycin ((**A**) internal standard, IS) and lekethromycin (**B**).

To optimize the chromatographic conditions, several commercial reversed-phase columns were evaluated, including Acquity ethylene bridged hybrid (BEH) C18 (100 × 2.1 mm i.d. particle size = 1.7 μm), Phenomenex Kinetex C18 column (50 × 2.1 mm i.d. particle size = 2.6 μm), Luna Omega PS C18 column (30 × 2.1 mm, 3 μm) and Acquity BEH hydrophilic interaction chromatography (HILIC) column (50 × 2.1 mm i.d. particle size = 1.7 μm). Different mobile phases were also compared, including water–ACN and water–MeOH, which were paired with different strength buffer solutions: 0.1% FA, 0.5% FA and ammonium formate and ammonium acetate solutions. Ultimately, the Luna Omega PS C18 column (30 × 2.1 mm i.d. particle size = 3 μm) in combination with 0.5% FA in water–0.5% FA in ACN proved to be the most appropriate because it gave good peak shape and signal intensity, as well as short run time (3.5 min), which is suitable for routine analysis. The optimal gradient elution program at a column temperature of 35 °C was established, which resulted in not only satisfactory separation efficiency but also lower background noise. The mass spectra of the precursor to product ions [M + H]^+^ for lekethromycin and the IS are displayed in Figure 2.

ACN-mediated protein precipitation is a commonly used sample extraction method, which is simple, economic and timesaving. In our study, ACN was also chosen as the plasma protein precipitant since it was free from the matrix effect and gave good extraction efficiency.

### 3.2. Method Validation

#### 3.2.1. Specificity and Sensitivity

**Figure 3:**
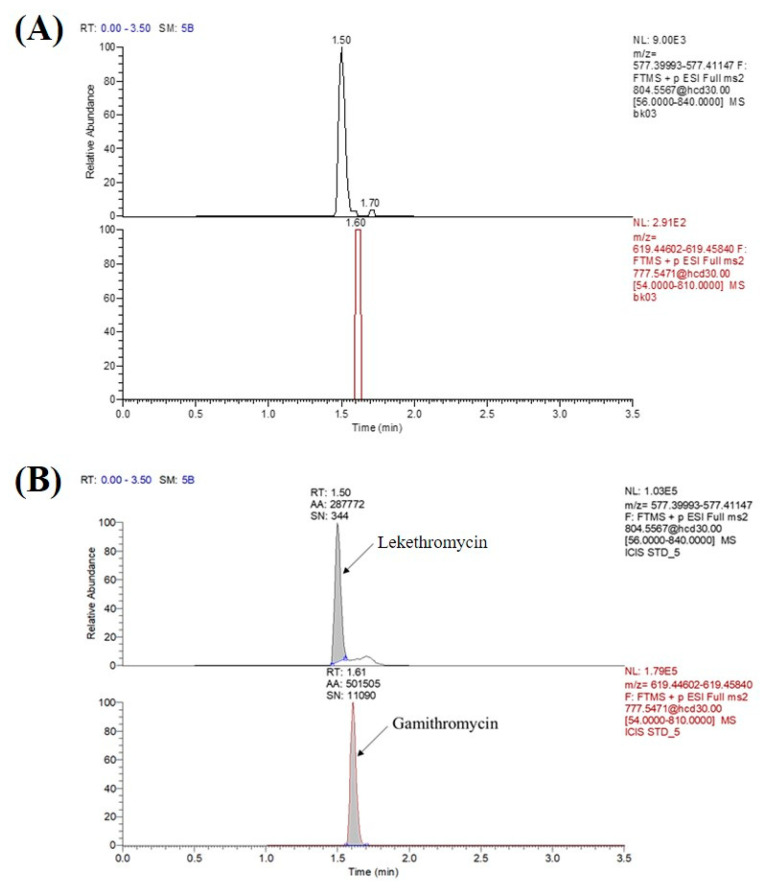
Chromatograms of lekethromycin and internal standard in blank rat plasma (**A**) and plasma spiked at lower limit of quantification (LLOQ) level (**B**).

Representative chromatograms of blank rat plasma sample (A) and blank rat plasma spiked with lekethromycin at the LLOQ (5 ng/mL, B) are illustrated in Figure 3. The results showed that the signal response of interfering components in the blank sample was <20% of the LLOQ for lekethromycin and <5% that of the IS, which met the requirement for bioanalytical method validation. The retention times of lekethromycin and IS were 1.5 and 1.61 min, respectively. The LLOQ, the lowest concentration that can be accurately quantified in the standard curve, was measured and determined as 5 ng/mL with *S/N* > 10, and the RSD (± 20%) was within the required limits. After ULOQ sample injection, the peak areas of the blank samples were <20% of the LLOQ for lekethromycin and <5% that of the IS, which suggested acceptable carryover effect.

#### 3.2.2. Linearity and Calibration Curve

A calibration standard curve with six points was constructed by plotting peak area ratios (lekethromycin/IS, Y) against nominal concentrations of lekethromycin (X) in the range of 5–1000 ng/mL and the typical regression equation was y = 0.267x + 0.148. For each analytical run, good linearity (r^2^ > 0.99) was observed with RSD < 15%.

#### 3.2.3. Precision and Accuracy

The intra- and inter-day precision and accuracy values are summarized in Table 1. Notably, precision values (RSD%) ranged from 5.5%–7.3% and 3.5%–6.3% for inter- and intra-day, respectively. The accuracy values for intra- and inter-day were 90%–94% and 93%–97%, respectively. The results demonstrated that the analytical method was accurate and reproducible and satisfied requirements.

#### 3.2.4. Recovery and Matrix Effects

The extraction recovery and ME results are presented in Table 2. The average recovery was greater than 95% ± 4.8%, with RSD < 5.1%, and the mean internal standard corrected matrix effect ranged from 0.96% to 1.0%, with RSD < 5.6%. The results indicated that the proposed method had good extraction efficiency and an acceptable matrix effect.

#### 3.2.5. Dilution Integrity

The dilution integrity results are presented in Table 3. The QC samples (2000 ng/mL) were diluted 2-, 10- and 100-fold and then analyzed. The accuracy for the analysis of the diluted samples was 99%–114%, with RSD < 3.2%. The results suggested that dilution of the plasma samples up to 100-fold did not affect the accurate determination of lekethromycin.

#### 3.2.6. Stability

The stability results of lekethromycin in rat plasma are shown in Table 4. Stability is reported as accuracy and precision relative to freshly prepared calibration plasma samples. It can be seen that accuracy under the five conditions was 92%–114%, with RSD < 12%. The results demonstrated that lekethromycin was stable in plasma at room temperature (approximately 25 °C) for 24 h, at –20 °C for three months, after three freeze and thaw cycles and at 7 °C for 8 h in the autosampler. Moreover, stock standard solutions (1 mg/mL) after appropriate dilution were also analyzed, and no significant degradation was observed after storage at –20 °C for three months.

### 3.3. Pharmacokinetic Study

**Figure 4:**
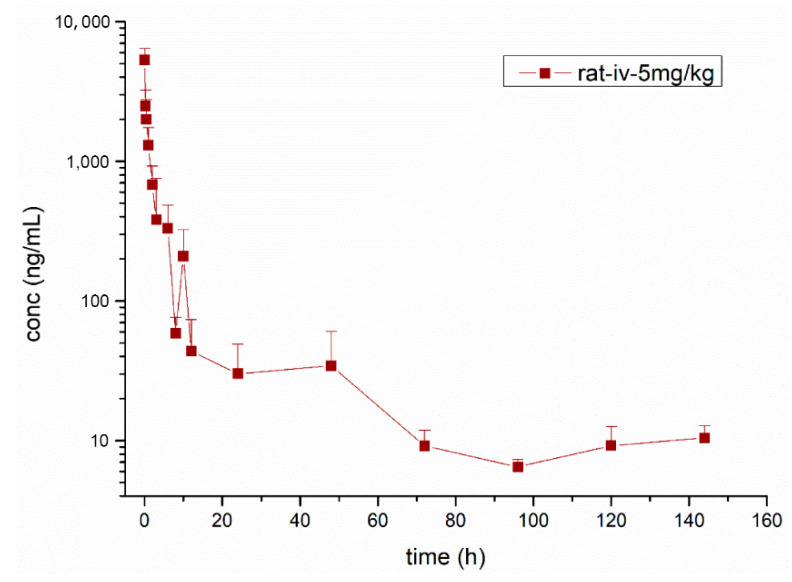
Mean plasma concentration-time profile of lekethromycin in rats after intravenous administration of lekethromycin at a single dose of 5 mg/kg.

The validated UPLC-ESI-Orbitrap-MS analytical method was fruitfully applied in a preclinical pharmacokinetic study in rats after intravenous administration of lekethromycin at a single dose of 5 mg/kg. Figure 4 shows the mean plasma concentration of lekethromycin versus time in rats. The main pharmacokinetic parameters calculated using a non-compartmental model are presented in Table 5. The results showed that lekethromycin was slowly eliminated from rat plasma after intravenous administration with T_1/2λz_ of 32.33 ± 14.63 h. Its clearance was determined to be 0.58 ± 0.17 L/h/kg, which was far below the blood flow (5.1 L/h/kg), indicating a low clearance rate and high plasma exposure in rat. The V_Z__obs and MRT_last_ were 25.56 ± 7.93 L/kg and 17.38 ± 7.71 h, respectively, suggesting that lekethromycin is widely distributed and has a long residence time in rats. The successful application of the analytical method to the preliminary pharmacokinetic study of lekethromycin indicated that the established method was reliable and suitable.

## 4. Conclusions

A rapid, highly sensitive and selective UPLC-ESI-Orbitrap-MS bioanalytical method was developed for the quantification of lekethromycin in rat plasma for the first time. The method has been fully validated for specificity and sensitivity, linearity, accuracy, precision, matrix effect, extraction recovery, dilution integrity and stability in accordance with FDA and EMA guidelines. The established method displayed major advantages in terms of simple sample preparation, fast chromatographic separation and high-throughput efficiency. This novel method has been fruitfully employed to study the pharmacokinetic profile of lekethromycin following intravenous administration of 5 mg/kg in rats. The results indicated that lekethromycin was widely distributed with low clearance pharmacokinetic behavior in rats, which may be valuable for future studies exploring the clinical pharmacokinetic profile of lekethromycin in subjects.

## Figures and Tables

**Table 1:** Inter- and intra-day precision and accuracy of lekethromycin in rat plasma.

Concentration (ng/mL)	Intra-day (n = 6)		Inter-day (n = 18)	
	Accuracy (%)	Precision (RSD%)	Accuracy (%)	Precision (RSD%)
15	94	5.5	97	5.1
200	95.	3.9	97	3.5
800	89.	7.3	93	6.3

**Table 2:** Recovery and matrix effect of lekethromycin in rat plasma.

Concentration (ng/mL)	Recovery (n = 6)		Matrix Effects (n = 6)	
	Accuracy (%)	Precision (RSD%)	Mean (%)	Precision (RSD%)
15	98	3.3	0.96	5.6
200	98	2.9	0.97	2.2
800	95	5.1	1.0	1.2

**Table 3:** Dilution integrity of lekethromycin in rat plasma (n = 6).

Dilution Factor	Nominal Conc (ng/mL)	Measured Conc (ng/mL)	Accuracy (%)	Precision (RSD%)
100	20	20	99	2.0
10	200	2.2 × 10^2^	1.1 × 10^2^	1.7
2	1000	9.7 × 10^2^	97	3.2

**Table 4:** Stability of lekethromycin under different storage conditions (n = 3).

Stability	Nominal Conc (ng/mL)	Measured Conc (ng/mL)	Accuracy (%)	Precision (RSD%)
Short-term (24 h)	15	15	1.0 × 10^2^	5.6
	200	2.1 × 10^2^	1.0 × 10^2^	3.3
	800	7.7 × 10^2^	96	7.7
Long-term (2 months)	15	15	1.0 × 10^2^	3.8
	200	2.2 × 10^2^	1.1 × 10^2^	8.2
	800	9.1 × 10^2^	1.1 × 10^2^	11.7
Thaw and freeze (3 cycles)	15	15	97	7.4
	200	2.0 × 10^2^	1.0 × 10^2^	5.3
	800	7.4 × 10^2^	93	7.0
Auto-samples (8 h)	15	15	98	5.2
	200	2.0 × 10^2^	100	3.1
	800	7.3 × 10^2^	92	4.9
Stock solution (3 months)	100	97	98	2.8

**Table 5:** Pharmacokinetic parameters of lekethromycin after intravenous administration (5 mg/kg) in rats.

Parameters	Unit	Mean ± SD
λz	H^−1^	0.02 ± 0.01
T_1/2λz_	h	32.33 ± 14.63
C_max_	ng·mL^−1^	5735.97 ± 1395.96
AUC_last_	ng·h·mL^−1^	8710.69 ± 2318.88
AUC_INF__obs	ng·h·mL^−1^	9133.46 ± 2372.71
V_Z__obs	L·kg^−1^	25.56 ± 7.93
CL_obs	L·h^−1^·kg^−1^	0.58 ± 0.17
MRT_last_	h	17.38 ± 7.71

λz, the elimination rate constant; T_1/2λz_, elimination half-life; C_max_, plasma peak concentration; AUC_last_, area under the concentration-time curve from 0 to the last point; AUC_INF_obs_, area under the concentration-time curve from 0 to infinity; V_Z__obs, apparent volume of distribution; CL_obs, apparent body clearance; MRT_last_, mean residence time.
